# Lysosomal ion channels and pain

**DOI:** 10.1097/PR9.0000000000001282

**Published:** 2025-06-05

**Authors:** Wanxue Liu, Yiming Li, Yuhan Bao, Zhi-Yong Tan

**Affiliations:** a School of Basic Medicine, Hebei University, Baoding, Hebei, China; b Stark Neurosciences Research Institute, Indiana University School of Medicine, Indianapolis, IN, USA; c Key Laboratory of Aging and Health in Hebei Province, Baoding, Hebei, China

**Keywords:** Lysosomal ion channels, Pain, Tmem63A, P2X4, TRPM8

## Abstract

Lysosomes are recycling centers of nearly all types of eukaryotic cells. Lysosomal ion channels maintain ion homeostasis of lysosomes and exchange ions with neighboring cytoplasm and subcellular structures. In these ways, lysosomal ion channels contribute to major function of lysosomes such as autophagy and lysosomal exocytosis. Deficiency in some lysosomal ion channels results in lysosome storage disorders such as mucolipidosis IV that is associated with early-onset neurodegeneration. Moreover, lysosomal ion channels are involved in a variety of conditions such as cancer, infectious diseases, respiratory diseases, cardiovascular and kidney diseases. This narrative review aims to summarize current evidence that supports the potential role of lysosomal ion channels in pain. Lysosomal P2X4 may contribute to pain through trafficking to plasma membrane as well as lysosomal exocytosis. In dorsal root ganglion neurons, lysosomal TRPM8 functions as a constitutive supply from lysosomal to plasma membrane, whereas lysosomal TRPA1 may mediate vehicle exocytosis of neurotransmitters. Moreover, recent studies suggest that Tmem63A forms a mechanosensory ion channel in lysosomal membrane and that Tmem63A of dorsal root ganglion neurons contributes to mechanical hypersensitivity in chronic pain models. Furthermore, evidences indicating a potential role of TRPMLs in pain include ROS sensitivity of TRPML1, chemokine release mediated by TRPML2, and re-expression of TRPML3 upon nerve injury. However, despite the current supporting evidence, the role of lysosomal ion channels in pain is just being explored, and future studies are needed to address the significance, mechanism, and potential translation of lysosomal ion channels in pain.

## Abstract 2

The role of lysosomal ion channels in pain is being explored. Evidence so far indicates an important role and multiple mechanism involved.

## 1. Lysosomal function, ion regulation, and lysosomal ion channels

Lysosomes are recycling centers of most mammalian cells. Through pathways of endocytosis and autophagy, lysosomes receive substrates from outside and inside of cells, respectively.^110,145^ Within lysosomes, macromolecules are broken down to amino acids, monosaccharides, and free fatty acids by lysosomal hydrolases.^1,8^ The products of lysosomal degradation are transported out of lysosomes back to the cytosol or outside of cells.^109,111,145^ In addition to intracellular degradation, secreted lysosomal hydrolases are involved in the degradation of extracellular material such as bones or pathogens.^71,89^ Furthermore, interaction with plasma membrane or other organelles enable lysosomes to exhibit nondegradation functions such as membrane repair, release of functional molecules, and regulation of other intracellular component such as mitochondria and ER.^3,9,108,128^

**Figure 1.**
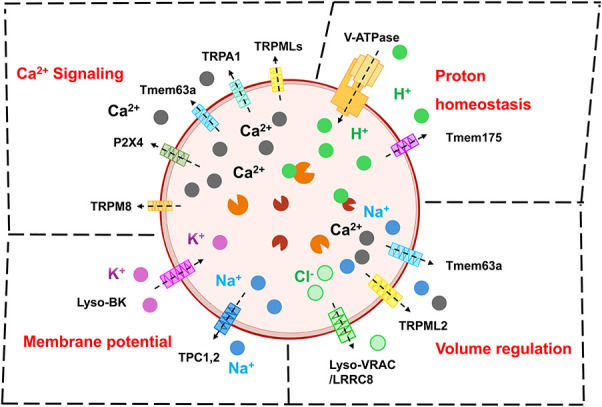
Lysosomal ion channels and ion regulation. Selected lysosomal ion channels and their associated role in lysosomal ion regulation. Top-Right: Tmem175 and V-ATPase maintain lysosomal proton homeostasis that is critical for lysosomal hydrolases and exporters to degrade substrates and recycle products, respectively. Top-Left: Multiple Ca^2+^-permanent channels mediate Ca^2+^ flux across lysosomal membrane that triggers lysosomal trafficking, autophagy, lysosomal exocytosis, organelle crosstalk, and lysosomal biogenesis. Down-Left: Sodium and potassium channels (such as TPCs and Lyso-BK) are major determinants of lysosomal membrane potential that modulates lysosomal ion currents and homeostasis. Down-Right: Multiple mechano/osmo-sensitive ion channels may be involved in the volume/size regulation of lysosomes.

Ion homeostasis and ion channels are essential for lysosomes to function. Particularly, a high proton environment of pH = 4 to 5 is critical for most of lysosomal hydrolases to digest their substrates. Moreover, the proton gradient across lysosomal membrane facilitates the transport of soluble products from lysosomal lumen to the cytosol by specific exporters located in the lysosomal membrane.^61,159^ The proton homeostasis of lysosomes is maintained by 2 major mechanism via 2 types of lysosomal membrane proteins: vacuolar H^+^-ATPase (V-ATPase) that pumps protons into the lysosomal lumen^23,84^ and the Tmem175 channel that conducts a proton flux out of lysosomal lumen (Fig. 1).^51^

Similar to proton, lysosomes have a higher Ca^2+^ concentration compared to the cytosol. It is estimated that the Ca^2+^ concentration is approximately 0.5 mM in lysosomal lumen, around 5,000 times higher than the cytoplasmic level.^9,50^ A Ca^2+^ flux from lysosomal lumen to the cytosol is critical for lysosomal function such as lysosomal trafficking, fusing with endosomes (in the endocytosis process), fusing with autophagosomes (in the autophagy flux), lysosomal exocytosis, organelle crosstalk, and lysosomal biogenesis.^13,20,108^ Accordingly, multiple Ca^2+^-permanent ion channels are located in the lysosomal membrane including TRPMLs (TRPML1-3), TRPA1, TRPM8, Tmem63A, and P2X4 (Fig. 1).^15,50,134^ Moreover, TPC2 can permeate Ca^2+^ under certain conditions.^38,44^ Among them, TRPMLs are preferentially expressed in the endolysosomal system, whereas TRPA1, TRPM8, and P2X4 can also express in the plasma membrane. Particularly, TRPML1 is selectively expressed in the late endosomes and lysosomes (LELs) and is considered as the principle Ca^2+^-permanent channel in the lysosomal membrane.

The membrane potential of lysosomes is an important regulator of lysosomal ion flux and ion homeostasis. For example, the lysosomal membrane potential modulates the amount of H^+^ or Ca^2+^ passing through Tmem175 or TRPML1, respectively. Considering the amount of ion concentration across lysosomal membrane, Na^+^ (50–140 mM in lysosomes vs 15 mM in the cytosol) and K^+^ (10–30 mM in lysosomes vs 150 mM in the cytosol) may be the major contributors to lysosomal membrane potential (Fig. 1).^50^ Na^+^ flux from lysosomal lumen to the cytosol and K^+^ flux from the cytosol to lysosomal lumen depolarizes and hyperpolarizes lysosomal membrane, respectively. TPCs (TPC1-2) are endolysosomal Na^+^ channels, and TPC2 is selectively expressed in LELs.^44,137^ On the other hand, Lyso-BK may be the major potassium channel in the lysosomal membrane.^34,143^

Lysosomes change their size/volume under certain conditions. For example, nutrient starvation triggers the fusion of lysosomes and the increase of lysosomal diameter by 3 to 5 times.^145^ Moreover, hypoosmolarity swells lysosomes. Lysosomes express multiple mechano/osmo-sensitive ion channels including LRRC8, TRPML2, and Tmem63A (Fig. 1).^16,73,74^ Upon activation by hypoosmolarity, LRRC8 mediates anion currents, whereas TRPML2 and Tmem63A mediate cation currents across lysosomal membrane.

## 2. Lysosomes and pain

The function of lysosomes (such as protein degradation and related protein turnover) is essential for sensory neurons, especially for dorsal root ganglion (DRG) neurons that have extremely long axons. To overcome the long axon–derived challenge of maintaining cellular homeostasis, soma of DRG neurons continuously deliver lysosomes to distal axons to maintain local degradation capacity.^31,47^ Lysosomes are positioned along axons and enriched at distal terminals of DRG neurons. Inhibition of axonal delivery of lysosomes induces aberrant accumulation of autophagosomes in distal axons of DRG neurons. Moreover, reduced function of lysosomes, as seen in multiple lysosomal storage disorders, results in the pathological alterations in the axons and soma of DRG neurons.^35,48,62,112^

**Figure 2.**
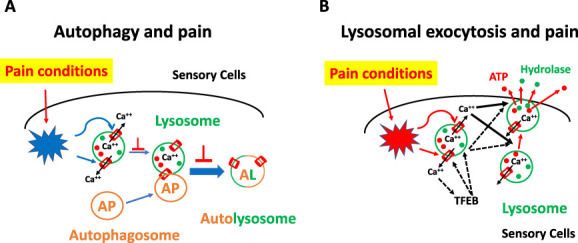
Lysosome ion channels may contribute to pain through autophagy or lysosomal exocytosis. (A) Pain insults activate lysosomal cation ion channels and promote autophagy that may relieve pain; (B) Pathological activation of lysosomal cation ion channels may increase pain through lysosomal exocytosis.

It has been reported that lysosome-involved function of autophagy and lysosomal exocytosis is closely related to pain (Fig. 2). Autophagy is an essential function of most mammalian cells. Cells digest unnecessary or dysfunctional macromolecules and organelles and recycle useful component through autophagy. Studies have suggested that autophagy plays an important role in chronic pain (Fig. 2A). Most of these studies suggest that autophagy is impaired in sensory neurons and/or glia of peripheral nervous system and/or spinal cord in rodent models of chronic pain.^7,76^ Some studies suggest that the baseline autophagy is actually enhanced in some of these cells.^81^ However, no matter how the baseline autophagy activity is altered, almost all the studies have found that activation of autophagy inhibits while inhibition of autophagy increases neuropathic pain behaviors.^7,76,81^ Therefore, autophagy negatively contributes to chronic pain in general (Fig. 2A).

In addition to autophagy, some studies suggest that lysosomal exocytosis of ATP from peripheral sensory cells contribute to neuropathic pain induced by peripheral nerve injury. Jung et al.^59^ have studied the potential role of lysosomal exocytosis of ATP from DRG neurons. In an earlier study, they have found that ATP and Lamp1 are colocalized in cell bodies and distal neurites of dissociated DRG neurons. ATP release from DRG neurons, induced by extracellular ATP or zymosan, is reduced by inhibitors of lysosomal exocytosis. These results suggest that ATP is stored in the lysosomes of DRG neurons, and it is released from DRG neurons through lysosomal exocytosis. In a later study, Jung et al.^60^ have further found that ATP and Lamp1 are colocalized in the sensory projections of spinal cord dorsal horn. Spinal nerve ligation largely increased the level of intracellular ATP and the expression of Lamp1 without changing their colocalization rate in spinal cord dorsal horn. Moreover, the authors have suggested that ATP is anterogradely transported to dorsal horn from soma of DRG neurons. Overall, it is suggested that increased lysosomal exocytosis of ATP at cell body and/or nerve termini of DRG neurons might contribute to neuropathic pain associated with peripheral nerve injury. Similar to DRG neurons, lysosomal exocytosis of ATP from Schwann cell may contribute to HIV-induced neuropathic pain.^25^ HIV gp120 induces lysosomal exocytosis of ATP from Schwann cells, which results in the calcium increase and ROS generation in DRG neurons. Taken together, lysosomal exocytosis may positively contribute to chronic pain in general (Fig. 2B).

## 3. Lysosomal ion channels and pain

### 3.1. P2X4: function in both lysosomal and plasma membrane

Among P2X receptors, P2X4 is most widely distributed across tissues.^92,125^ In contrast to other P2X receptors, P2X4 is mainly expressed in endosomes/lysosomes, and there is a low level of surface expression of P2X4 under normal condition.^106^ Although there is a high level of ATP stored within lysosomes, the acidic environment of lysosomes generally prevents P2X4 from activation by ATP. Accordingly, deacidification of lysosomes results in the activation of P2X4 by ATP, which contributes to endolysosomal fusion and vacuolation.^86^ On the other hand, lysosomal P2X4 may also be activated without lysosomal deacidification, which promotes degradation, autophagy, lysosomal exocytosis, and phagocytosis.^53,75,127,152^

**Figure 3.**
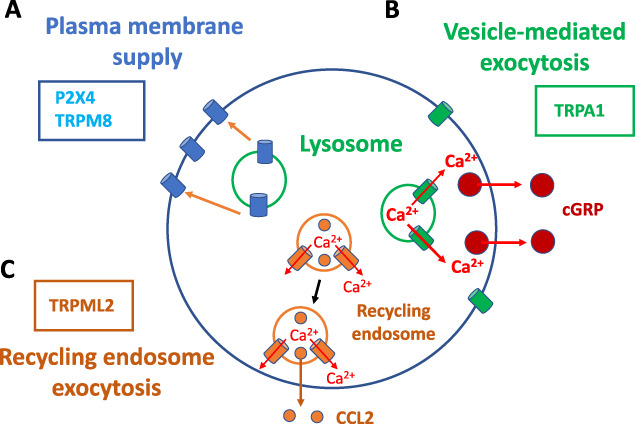
Other mechanisms underlying endolysosome ion channels and pain. Endolysosomal ion channels may contribute to pain through trafficking to the plasma membrane (A), vesicle-mediated exocytosis (B), and exocytosis from recycling endosomes (C).

P2X4 plays a key role in chronic pain. For example, P2X4-mediated BDNF release from spinal microglia or sensory neuron projection is important mechanism for chronic pain.^30,125^ Presumably, both increased de novo expression and lysosome–plasma membrane trafficking of P2X4 would contribute to P2X4-mediated chronic pain. In fact, spinal microglial P2X4-mediated nerve injury pain is likely contributed to by CCL2-induced trafficking of P2X4 from lysosomes to plasma membrane of microglial cells.^130,132^ Moreover, lysosomal P2X4 and VNUT might be activated by lysosomal deacidification induced by HIV gp120 in Schwann cells.^25^ Activated P2X4 and VNUT contribute to gp120-induced lysosomal exocytosis of ATP from Schwann cells, which increases intracellular calcium and ROS in DRG neurons. These results suggest that lysosomal P2X4 may contribute to chronic pain through 2 different cellular mechanism: trafficking to and being activated in plasma membrane and mediating calcium influx from outside to inside of cells; being activated in lysosomal membrane and promoting lysosomal exocytosis from inside to outside of cells (Figs. 2B and 3A).

### 3.2. TRPM8: constitutive supply from lysosomal to plasma membrane

TRPM8 is a member of the TRPM (Melastatin) family.^29^ TRPM8 is broadly expressed in peripheral tissues through innervation of TRPM8-expressing sensory neurons and/or the additional expression in tissue cells.^78,82,98^ These tissues include skin, cornea, airway, colon, bladder, prostate, immune cells, and sperm. A TRPM8 channel is composed of 4 functional subunits (homotetramer).^57^ Each functional subunit consists of 6 transmembrane segments (S1-6), and large intracellular domains at both carboxyl and amino termini. Similar to other voltage-gated ion channels, S1-4 is the voltage sensor domain while S5-6 is the pore-forming domain.^151^

TRPM8 is polymodally gated ion channel that is activated by multiple stimuli.^40,54^ These stimuli include nociceptive to innocuous cold, cooling agents, membrane depolarization, and hyperosmolarity. Activation of TRPM8 results in an inward, calcium-permeable cation current and membrane depolarization that may contribute to the initiation of action potentials (through the generation of the transient receptor potential), release of neuroactive chemicals, and cell signaling in sensory neurons. The activity of TRPM8 is modulated by a variety of physiological factors. For example, PI(4,5)P2 positively modulate TRPM8.^107^ The depletion of PI(4,5)P2 by phospholipase C is a common mechanism for the desensitization of TRPM8 after the channel activation and calcium influx.

TRPM8 is involved in a variety of physiological function and, therefore, pathological conditions. These conditions include pain, migraine, cancer, dry eye, bladder hyperactivity, and respiratory dysfunction.^85,93,119,150^ In the peripheral sensory system, TRPM8 is selectively expressed in a subset of primary sensory neurons that are cold-sensitive. It has been well established that TRPM8 function as a major peripheral cold sensor for both innocuous and nociceptive cold stimuli. Accordingly, TRPM8 is critical in cold hypersensitivity seen in multiple neuropathic pain conditions. For example, in DRG neurons, TRPM8 is acutely activated in vitro by a chemotherapeutics oxaliplatin and the expression of TRPM8 is upregulated after oxaliplatin treatment in vivo.^88,139,148^ Both TRPM8 knockout and antagonists reduce oxaliplatin-induced cold hypersensitivity.^36,90,139^

It has not been clear that how the intracellular pool supplies plasma membrane with TRPM8. A couple of recent studies suggest that LEL is the intracellular TRPM8 pool supplying plasma membrane constitutively and upon mobilization. Ghosh et al.^39^ have reported that in vitro expressed TRPM8 (in HEK293) is selectively coexpressed with markers of lysosomes (Lamp1) and late endosomes (Rab7), but not early endosomes (EEA, Rab4), recycling endosomes (Rab11), vesicle-mediated endocytosis and/or exocytosis (caveolin and clathrin), ER (VSVG and SERCA), and Golgi apparatus (SPCA). These results suggest that in vitro overexpressed TRPM8 is selectively expressed in LEL but not other intracellular component. Furthermore, reduced transfection of TRPM8 does not change the colocalization of TRPM8 with Lamp1. Lamp1 selectively colocalizes with TRPM8 and TRPM3, but not TRPV1, TRPA1, TRPV2, and TRPM4. These results suggest that the expression of TRPM8 in lysosomes is specific and not due to an overexpression that results in the lysosomal degradation of TRPM8. Interestingly, among Lamp1-positive puncture-like structures, TRPM8 is selectively expressed with those of not or less acidic, near the plasma membrane, but not those of acidic and distal from plasma membrane. These results suggest that TRPM8 is selectively expressed in the lysosomes that are close to and would fuse with plasma membrane, but not those near nucleus and would break down unwanted macromolecules with an acidic lumen. In fact, using TIRF to study the recovery from photobleaching, the authors have found that there is a constitutive transport of TRPM8 from Lamp1-positive vesicles to plasma membranes. These results suggest that lysosomes constitutively supply plasma membrane with TRPM8. Moreover, VAMP7 plays a critical role in facilitating the transport of functional TRPM8 to plasma membrane.

In addition to the in vitro expression of TRPM8 in HEK293 cells, the authors have further provided relevant evidence in trigeminal ganglion (TG).^39^ The evidence includes colocalization of TRPM8 and Lamp1 in neurites of TG neurons, transport of TRPM8 and Lamp1 positive structure to the tip of neurites, and reduction of functional TRPM8 in plasma membrane of TG neurons by VAMP7 knockout. These results suggest that a constitutive transport of TRPM8 from lysosomes to plasma membrane may occur in vivo in axon and/or cell body of primary sensory neurons. To support this suggestion, the authors have found that VAMP7 knockout reduces acute cold avoidance and icilin-induced cold responses in mice. Overall, it is suggested that a constitutive transport of functional TRPM8 from lysosomes to plasma membrane in primary sensory neurons maintains the homeostasis of plasma membrane TRPM8, which may potentially contribute to TRPM8-mediated cold sensitivity.

Along with the above report, another study from Kayama et al.^64^ suggests that NGF can increase TRPM8 trafficking to plasma membrane from lysosomes. Using a stable expression PC12 cell line, the authors have found that NGF increases colocalization of TRPM8 with Lamp2 (a lysosomal marker) at cell periphery and plasma membrane. These results suggest that a lysosome–plasma membrane trafficking mediates NGF-induced upregulation of plasma membrane TRPM8. Furthermore, the authors have found that the increasing effect on plasma membrane TRPM8 is mediated by NGF-TrK A-PI3K-p38MAPK signaling pathway. In addition to the incorporation of TRPM8 into plasma membrane, the authors also studied degradation of TRPM8. Interestingly, they have found that TRPM8 degradation is mediated by proteosome pathway but not the lysosome-mediated autophagy pathway. These results suggest that NGF enhances TRPM8 trafficking from lysosomes to plasma membrane and lysosomes do not degrade TRPM8. As NGF-TrK A-PI3K-p38MAPK plays important role in pathological pain conditions, these results also suggest that lysosomal trafficking TRPM8 to plasma membrane may contribute to pathological pain conditions.

Taken together, these studies suggest that lysosomes can supply TRPM8 to plasma membrane under normal or stimulated conditions in primary sensory neurons (Fig. 3A). The lysosome–plasma membrane trafficking of TRPM8 may contribute to acute cold sensitivity and cold hypersensitivity under pathological pain conditions such as cancer therapy, diabetes, spinal cord injury, viral infection, multiple sclerosis, or withdrawal symptoms associated with chronic morphine treatment. In addition to supply plasma membrane, lysosomal TRPM8 may directly affect lysosomal function.^118^ For instance, in a microglial cell line, inhibition of TRPM8 selectively acidifies lysosomes but not the cytosol, whereas activation of TRPM8 reduces LPS-induced lysosomal acidification. These results suggest that activation of lysosomal TRPM8 may promote antibacterial or anti-inflammation effects of microglia through deacidification of lysosomes.

### 3.3. TRPA1: vesicle-mediated exocytosis induced by lysosomal calcium release

TRPA1 is the only mammalian member of the TRPA (ankyrin) family. TRPA1 is broadly expressed in peripheral tissues (through innervation of the primary sensory neurons and/or in epithelial tissue cells) that involves in function of near all the organs.^83,126^ On top of the overall structure of individual subunit and tetrameric channels similar to other TRP channels, there is a 14 ankyrin repeats located in the NH2 terminus of the TRPA1 channels. TRPA1 is a nonselective cation channel and is activated by a variety of noxious stimuli including intense cold,^124^ pungent compounds,^6^ and reactive chemical species.^96^ Particularly, TRPA1 is activated by an unusually wide variety of compounds.^52^

TRPA1 is expressed in nociceptive DRG neurons.^4,80,121^ Due to its diverse activation mechanism, TRPA1 functions as a polymodal nociceptive sensor for cold, mechanical force, oxidative and nitrosative stress, and diverse natural compounds and other xenobiotics.^2,70,91,135^ Accordingly, TRPA1 is broadly involved in a variety of pathological pain conditions.^12,19,49,63,69,90,95,123,131^

Compared to the plasma membrane TRPA1,^5,115^ the role of intracellular TRPA1 has not been well studied. A couple of studies suggest that TRPA1 may be expressed in the lysosomal membrane and contribute to release of neurotransmitters in DRG neurons. Shang et al.^117^ have first reported a role of lysosomal TRPA1 in increasing cytoplasm calcium and vesicle-mediated exocytosis of neurotransmitters. Using calcium imaging, these authors have found that TRPA1-mediated increase in the intracellular calcium is not completed blocked by the depletion of external calcium or by membrane-impermeable TRPA1 blockers. However, the TRPA1-mediated calcium response is completed blocked by TRPA1 knockout or by a membrane-permeable TRPA1 inhibitor. These results suggest that functional TRPA1 is expressed intracellularly and activation of intracellular TRPA1 increases intracellular calcium.^117^ Moreover, the intracellular TRPA1-mediated calcium response is selectively blocked by inhibitors of lysosomes, but not inhibitors of ER or mitochondria. TRPA1 is colocalized with Lamp1. These results suggest that functional TRPA1 is selectively expressed in lysosomes, and activation of lysosomal TRPA1 increases intracellular calcium. Furthermore, the authors have found that lysosomal TRPA1 contributes to the vesicle-mediated exocytosis of CGRP in DRG neurons. Altogether, these results suggest that functional TRPA1 is expressed in lysosomes and contributes to increase in intracellular calcium and subsequent vesicle-mediated exocytosis of neurotransmitters in DRG neurons (Fig. 3B). Therefore, lysosomal TRPA1 may potentially contribute to a variety of physiological and pathological pain conditions.

In a later publication, Gebhardt et al.^37^ also tested if intracellular or lysosomal TRPA1 contributes to intracellular calcium increase and vesicle-mediated exocytosis of neurotransmitters. The authors have firstly demonstrated that external calcium is necessary for trachea (contains innervation of sensory neurons) to release CGRP upon the activation of TRPA1 by AITC. Combined with previous studies on the sensory projection in the spinal cord, it is suggested that external calcium is necessary for release of CGRP from both peripheral and central nerve termini of sensory neurons upon activation of TRPA1. Furthermore, the authors reexamined the key calcium imaging experiments conducted by Shang et al., using cell bodies of dissociated DRG neurons. To deplete external calcium, the authors used 10 mM or 1 mM EGTA to buffer external solution (through 10 minutes bath exchange), different from the 1 mM EGTA (through puffing) in the earlier study conducted by Shang et al. It is found that AITC does not induce calcium rise in DRG neurons when external solution is buffered by 10 mM EGTA. On the other hand, AITC does induce intracellular calcium release in some DRG neurons when external solution is buffered by 1 mM EGTA. However, the response rate is much lower (14%–6%) compared to the previous study of Shang et al. (30%). Considering that 1-mM EGTA may not sufficiently buffer external calcium, the authors suggest that activation of TRPA1 does not induce calcium release from intracellular stores. Because the 10-minute washout of external calcium by EGTA might deplete the internal calcium stores, the authors also utilized a custom-made miniature dish to achieve the fast calcium depletion (<1 second). Using this fast exchange system, similar results are observed. These results suggest that the lower response rate (with 1 mM EGTA buffer) or lack of response (with 10 mM EGTA buffer) is due to reduction or depletion of external calcium, respectively, but not depletion of intracellular calcium stores. Overall, the authors suggest that AITC does not increase intracellular calcium or CGRP release through activation of intracellular TRPA1 in DRG neurons.

In response to the report from Gebhardt et al., Liu et al.^77^ have defended their major findings. First, the free calcium concentration in the nominal calcium-free bath solution with 1-mM EGTA is <4 nM, 10 times lower than intracellular calcium concentration (about 50 nM) of DRG neurons. And AITC, but not membrane depolarization, selectively increases intracellular calcium with 1 mM EGTA in the external solution. This evidence argues against a significant contribution of contaminated calcium from other regular chemicals or from loosely attached calcium ions on the plasma membrane, to the AITC-increased intracellular calcium with 1 mM EGTA in the external solution. Moreover, the authors suggest that the loss of intracellular TRPA1-mediated calcium response with 10-mM EGTA in the external solution could be resulted from the transport of extracellular EGTA to the inside of cells. Particularly, extracellular EGTA can be transported to lysosomes through endocytosis. Moreover, EGTA might pass through TRPA1 in the calcium-free condition. Both lysosomal or cytoplasm EGTA could prevent the AITC-induced calcium release from lysosomes. Similarly, the authors also suggest that the EGTA translocation from outside to inside of axons might interfere with the release of CGRP from termini of DRG neurons. Compared to cell body, the nerve termini may have a high surface-to-volume ratio and, therefore, higher capability to transport EGTA across plasma membrane. Overall, the authors suggest that lysosomal TRPA1 mediates calcium release from lysosomes that contributes to vesicle-mediated exocytosis of neurotransmitters from DRG neurons (Fig. 2B). The transport of EGTA across plasma membrane may confound with relevant experiments.

### 3.4. Tmem63A: a lysosomal mechanosensory channel for mechanical pain

Transmembrane protein 63A (Tmem63A) is a member of evolutionarily conserved family of mechanically activated ion channels (OSCA/Tmem63).^18,87^ Similar to the other family members of Tmem63B and Tmem63C, monomer of Tmem63A forms a high-threshold mechanosensitive, nonselective cation channel.^142,157^ Tmem63A is generally expressed across tissues. In central nervous system, it is highly expressed in the oligodendrocytes. Accordingly, heterozygous loss-of-function mutants of Tmem63A is associated with infantile-onset transient hypomyelination in human.^149^ In addition, Tmem63A is expressed in the pulmonary alveolar type 2 epithelial (AT2) cells.^17^ Together with Tmem63B, Tmem63A mediates lung inflation–induced surfactant secretion.

Proteomics studies have indicated that Tmem63A, but not Tmem63B or Tmem63C, is a lysosomal membrane protein in human, mouse, and rat cells.^14,101,116^ A recent study has confirmed the preferential expression of Tmem63A in lysosomes of mouse Neuro-2a cells.^73^ Moreover, the same study has first reported the recording of Tmem63A-mediated mechanosensitive currents in lysosomes of Neuro-2a cell. Furthermore, Drosophila Tmem63 (DmTmem63), but not other Drosophila mechanosensitive channels (Drosophila Piezo, NompC, Iav), is preferentially expressed in lysosomes, but not mitochondria or peroxisomes.^73^ In vitro expression experiments suggest that plasma membrane DmTmem63 forms a high threshold, nonselective cation, and low single-channel conductance mechanosensitive channel. Moreover, lysosomal DmTmem63 conducts lysosomal calcium flux upon hypotonic stimulation in the absence of extracellular calcium. In endogenous larva fat body cells, knockout of DmTmem63 largely reduces the mechanosensitive currents in lysosomes but not affects the mechanosensitive currents in the plasma membrane at all. Furthermore, DmTmem63 knockout increases size of lysosomes, reduces motor function, and shorten the lifespan of adult fly. These results first confirm that mouse Tmem63A and DmTmem63 form mechanosensitive ion channels in the lysosomal membrane, and they are important in lysosomal and systemic function.

A study of single-cell RNA-seq of mouse DRG neurons have found that Tmem63A is selectively expressed in 1 type of nociceptive neurons (nonpeptidergic).^133^ Using in situ hybridization, a recent study has confirmed this expression selectivity.^103^ Earlier transcriptome studies have found that the expression of Tmem63A in DRG is upregulated after peripheral nerve injury induced by spinal nerve ligation or a chemotherapeutics paclitaxel.^45,68^ Moreover, the expression of Tmem63A is upregulated under a neuropathic (tibial nerve transfer) and an inflammatory pain (CFA) conditions.^103^ Mouse knockout of Tmem63A selectively prevents mechanical but not thermal pain behaviors in these pain models.^103^ These results suggest that Tmem63A of DRG neurons may play an important role in mechanical allodynia under chronic pain conditions. Considering the aforementioned evidence for the expression of Tmem63A in lysosomes of multiple other cell types, it may be suggested that lysosomal Tmem63A of DRG neurons contribute to chronic pain. However, current evidence can not rule out a contribution from plasma membrane Tmem63A to pain behaviors under these conditions.

The lysosomal mechanism of Tmem63A in pain has not been reported. However, overexpression of DmTmem63 increases the peripheral positioning of lysosomes in *Drosophila* fat body cells that might promote lysosomal exocytosis.^73^ Considering that there is a positive correlation between Tmem63A and pain,^103^ and that lysosomal exocytosis is positively correlated to pain (Fig. 2B), it might be suggested that Tmem63A contributes to chronic pain through lysosomal exocytosis in nociceptive DRG neurons (Fig. 2).

### 3.5. TRPML1: lysosome-specific calcium-conducting channel activated by ROS

TRPML1 is 1 of the 3 members of the TRPML (mucolipin) family. TRPML1 is broadly expressed across tissues. Compared to other families of TRP channels, TRPMLs are preferentially expressed in the endolysosomal system.^108,134,145^ Compared to other members of the TRPML family (TRPML2 and TRPML3), TRPML1 is selectively expressed in the LEL. Similar to other TRP channels, TRPML1 channels are tetrameric and each subunit is composed of a S1–S6 transmembrane domains and intracellular loops at the N- and C-termini. Two di-Leucine motifs located, respectively, at the N- and C-terminal are important in the selective targeting of TRPML1 to LEL. TRPML1 is a calcium-permeable, nonselective cation channel. It is activated by a lysosomal lipid PI(3,5)P2 and inhibited by 2 plasma membrane lipids PI(4,5)P2 and sphingomyelin.

Zhang et al.^156^ have examined the effects of ROS on TRPML1. Using whole-lysosome patch clamp, they have found that TRPML1 currents are activated by multiple oxidants including chloramine T (ChT), NaOCl, N-chlorosuccinimide, thimerosal, H_2_O_2_ and t-butyl hydroperoxide (TBHP). On the other hand, TRPML1 is not activated by other oxidants including DTNP, DTNB, SNAP, and 4-HNE. Furthermore, utilizing a TRPML1 mutant that is expressed in the plasma membrane, they conducted the inside-out patch clamping and found that ChT activates TRPML1 currents in a plasma membrane patch from the cytoplasm site. These results suggest that ChT can directly activate TRPML1. In addition, the authors have found that the effects of ROS is selective for TRPML1 (but not for TRPML2, TRPML3, and TPC2) and common in multiple cell types (COS-1, HEK293, macrophage, HeLa, HGT-1, and HAP1). Overall, it is suggested that ROS directly and selectively activates TRPML1.

As a major lysosomal calcium-permeating ion channel, TRPML1 plays important roles in lysosomal function including ion transport, lysosomal trafficking, autophagy, and lysosomal exocytosis.^108,134,145^ Human mutants of TRPML1 are associated with type IV mucolipidosis, an early-onset neurodegenerative condition. Moreover, TRPML1 is also involved in other neurodegeneration conditions, cancer, muscular dystrophy, and lower urinary tract smooth muscle contractility.^21,41,43,120,141,146,147^ However, the role of TRPML1 in pain has not been reported. Considering that TRPML1 is ROS-sensitive and is important in both autophagy and lysosomal exocytosis, it would be interesting to study if TRPML1 positively contributes to pain through lysosomal exocytosis or if it negatively modulates pain via autophagy in sensory cells (Fig. 2).

### 3.6. TRPML2: mechano-sensitive endolysosomal channel and chemokine release

In contrast to a wide expression of TRPML1 across tissues, TRPML2 is expressed in restricted tissues including thymus, spleen, liver, kidney, and immune cells.^24,134^ Moreover, unlike the selective expression of TRPML1 in LEL, TRPML2 is expressed in the whole endolysosomal system including early endosomes (EE), late endosomes, lysosomes, and recycling endosomes (RE).

In a previous report, Plesch et al.^99^ have developed a selective agonist of TRPML2 (ML2-SA1) and have studied the function of TRPML2 in macrophage. First, using whole-endolysosomal patch clamp, they have demonstrated that ML2-SA1 activated inward currents in RE and/or LEL in LPS-activated bone marrow–derived macrophages (BMDM) or alveolar macrophages (AM). Then they have found that TRPML2 KO largely reduces LPS-induced release of CCL2 and that ML2-SA1 increases CCL2 release in wildtype but not TRPML2 KO BMDM after LPS pretreatment. These results suggest that endolysosomal TRPML2 contributes to the release of CCL2 in macrophages. Furthermore, the authors have found that ML2-SA1 does not increase the release of lysosomal hydrases nor the translocation of Lamp1 to plasma membrane but increases transferrin trafficking. These results suggest that ML2-SA1 increases CCL2 release through recycling endosomes but not lysosomal exocytosis (Fig. 3C). Considering macrophage infiltration and release of chemokines are common mechanism for chronic pain, macrophage TRPML2 might contribute to a variety of chronic pain conditions.

Using the same agonist of TRPML2 (ML2-SA1), the same group of researchers have studied how TRPML2 is activated in the endolysosomal system that results in the facilitation of endolysosomal trafficking.^16^ Using whole-endolysosomal patch clamp, the authors have found that hypotonic stimulation selectively activates TRPML2 currents, but not TRPML1 or TRPML3 currents in LELs of HEK293 cells, and TRPML2-like currents in LELs of activated macrophages. Because hypotonic stimulation would cause endolysosomal swelling and increase the mechanical tension of endolysosomal membrane, these results suggest that TRPML2 is a mechano-sensor of endolysosomal system. Furthermore, the authors have found that a leucine located at the lipid binding pocket is the key for the hypotonic activation of TRPML2. The hypotonic activation of TRPML2 may facilitate the fast recycling processes of immune cells. Overall, it is hypothesized that endolysosomal TRPML2 is activated by the mechanical tension produced by the accumulation of degraded molecules and subsequent swelling of endolysosomal system. Activation of TRPML2 reduces lysosomal cations, restores the size of endolysosomal system, and facilitates the endolysosomal trafficking.

### 3.7. TRPML3: Reexpression in dorsal root ganglion neurons upon peripheral nerve injuries

Compared to TRPML1 and TRPML2, TRPML3 is more restrictively expressed, mainly in hearing system and melanocytes.^42^ Similar to TRPML2, TRPML3 is expressed in the endosomes and lysosomes and regulates endocytosis, membrane trafficking, and autophagy.^67^ Moreover, TRPML3 may be expressed in the plasma membrane as well.^67^ Mouse gain-of-function mutants of TRPML3 (varitint-waddler mutations) are associated with deafness, circling behaviors, and coat color dilution.^113,122^

The overall biophysical properties of TRPML3 are similar to those of TRPML1 and TRPML2. However, TRPML3 is inhibited by low pH, whereas TRPML1 and TRPML2 are activated by low pH.^66,144^ In addition, TRPML3 appears to be relatively Fe^2+^-impermeable, whereas TRPML1 and TRPML2 are permeable to Fe^2+^.^28^

TRPML3 is normally expressed in DRG during embryonic development.^123^ However, a previous study has found that peripheral nerve injuries dramatically upregulate mRNA expression of TRPML3 in adult DRG neurons of rats and mice. For example, SNI increases the expression of TRPML3 by 77 and 65 times at 4 and 15 days postsurgery. The increased TRPML3 are expressed across all neuron subtypes tested (by size, or by neuronal markers of CGRP, NF200, and IB4). However, the absolute amount of TRPML3 transcript is still very low (0.3% compared to TRPV1) after nerve injury. Moreover, it is not clear whether the increased TRPML3 is located at plasma membrane or not (TRPML3 is presumably inhibited by the low pH of lysosomes). Therefore, further studies are needed to determine if DRG TRPML3 contributes to chronic pain.

### 3.8. Other endolysosomal ion channels

Tmem175 was initially reported as an endolysosomal potassium channel.^10^ However, a recent study suggests that Tmem175 is a proton-activated proton channel on the lysosomal membrane.^51^ Tmem175 is a genetic risk factor for Parkinson disease.^26,56^ However, both positive and negative contribution to Parkinson disease has been reported.^58,104^ Although a role of Tmem175 in pain has not been reported, Tmem175 is associated with neuroinflammation and other types of neurological diseases.^79,94,154,160^

The large-conductance, calcium-activated potassium channel (BK channel) is a type of voltage-gated potassium channels that features a dual activation mechanism by both membrane depolarization and cytoplasm calcium ions. A couple of studies have identified the functional expression of BK channels in the lysosomes (Lyso-BK) of a variety of cells.^11,136^ One of the studies suggests that activation of Lyso-BK channels hyperpolarizes lysosomal membrane and facilitates lysosomal calcium refilling from ER-lysosome contact sites.^136^ In contrast, the other study proposes that activation of Lyso BK enhances TRPML1-mediated lysosomal calcium release through hyperpolarizing lysosomal membrane.^11^ Accordingly to the latter study, BK agonists may rescue lysosomal storage disorders that are associated with neurodegeneration.^158^ In contrast to a general inhibitory role of BK channels in afferent pain neurons, spinal microglial BK channels positively contribute to chronic pain.^46,55^ Interestingly, a BK channel blocker paxilline suppresses the morphine-induced increase in plasma membrane P2X4 currents, which suggests that BK channels may regulate the membrane trafficking of P2X4 from lysosomes to plasma membrane in spinal microglia.^46^ It would be interesting to check if lysosomal BK channels might be involved in the P2X4 trafficking in spinal microglia under any pain conditions.

LRRC8 family members form a volume-regulated anion channel (VRAC) that is activated by hypoosmotic stimuli.^27,114^ In the plasma membrane, activation of VRACs produces outwardly rectifying anion currents that compensate for the swelling of cells induced by hypoosmotic stimuli.^97^ A recent study suggests that LRRC8 and VRACs are also expressed in the lysosomal membrane (Lyso-VRACs).^74^ Upon hypoosmotic stimuli, Lyso-VRACs mediate lysosomal vacuolation and exocytosis and reduces the swelling of cytoplasm of cells. Furthermore, Lyso-VRACs protect cell from necrosis under hypoosmotic, hypoxic, and hypothermic stresses. Interestingly, a recent study suggests that spinal microglial VRACs contribute to nerve injury–induced neuropathic pain through releasing ATP.^22^ As ATP is stored mainly in the lysosomes, it would be interesting to study if Lyso-VRACs may contribute to pain in this context or others.

Chloride channels/transporters are important in ion homeostasis of lysosomes. Deficiency of lysosomal chloride channel CLN7 is associated with a type of lysosomal storage diseases neuronal ceroid lipofuscinoses (NCL).^138^ Mouse knockouts of a lysosomal chloride transporter CLC-7, or a late endosome chloride transporter CLC-6, display neuropathologic phenotype of NCL.^102,153^ Although lysosomal chloride channels/transporters regulate lysosomal function through multiple mechanism, a couple of recent studies suggests that lysosomal chloride could also activates lysosomal acidic hydrolases.^33,140,155^ And at least CLC-7 contributes to a high intralysosomal chloride concentration. As CLC-6 knockout mice show reduced heat pain sensitivity, it would be interesting to examine if lysosomal CLC-7 and CLN7 also play a role in pain.^100^

### 3.9. Potential therapeutic targets for pain

Plasma membrane ion channels are a major type of therapeutic targets for diverse diseases including pain. With the advance in the knowledge of lysosomal ion channels, multiple of them have been considered as novel targets for neurodegenerative diseases.^65,105^ For examples, TRPML1 and TPC2 are considered promising druggable targets in neurodegenerative diseases that are partially due to defective autophagy and accumulation of toxic aggregates.^129^ Accordingly, agonists of TRPML1 and TPC2 may restore autophagy and reduce neurodegeneration in Parkinson, Alzheimer, and other diseases. Similarly, Tmem175 may be a potential target for Parkinson diseases.^32^

Similar to neurodegenerative diseases, lysosomal ion channels may be promising therapeutic targets for pain. First, lysosomal ion channels can be selectively targeted. For examples, some ion channels are preferentially expressed in the lysosomes (such as TRPML1, TPC2, and Tmem175). Moreover, conventional drugs with or without nanoparticles may be selectively delivered to lysosomes.^72^ Furthermore, the acid environment of lysosomes would allow development of acid-sensitive treatment. Second, for ion channels expressed in both lysosomal and plasma membrane (such as P2X4, TRPM8, and TRPA1), targeting both locations may increase the pain-relieving efficacy. On the other hand, selectively targeting lysosomal location may reduce side effects resulted from targeting plasma membrane. Third, tissue-specific expression (such as Tmem63A in IB4^+^ DRG neurons) would allow selective targeting of pain neurons.

## 4. Summary and conclusions

Lysosomal ion channels conduct ions (such as H^+^, Ca^2+^, Na^+^, K^+^, and Cl^−^) across lysosomal membrane to regulate/mediate lysosomal function including macromolecule degradation, export of soluble products, lysosomal trafficking, autophagy, lysosomal exocytosis, and volume regulation. Lysosomal function of autophagy and lysosomal exocytosis is inversely and positively associated with pain, respectively. Recent studies strongly suggest that multiple lysosomal ion channels (P2X4, TRPM8, TRPA1, Tmem63A, and others) contribute to pain through diverse mechanism including lysosomal exocytosis, supply to plasma membrane, vesicle-mediated exocytosis, and autophagy. However, the evidence so far is limited in the number of studies conducted and the integration of ion channel and pain in these studies. Particularly, future studies may need to focus on the contribution from lysosomes, ie, the role of lysosome-located ion channels (preferentially or proportionally) in pain. How important is it for each lysosome-located ion channel in pain? What kind of pain is involved (physiological or pathological? mechanical or thermal? neuropathic or inflammatory? diabetic, chemotherapy, or traumatic nerve injury)? Which type of cell is responsible for (sensory neurons, glia, or immune cells)? Mechanisms (causes of pain discussed in the current review and others)? Parallelly, therapeutic exploration of relevance to pain may be focused on lysosome-targeting in future studies. How to selectively target ion channels expressed in the lysosomes? How to develop small molecules with high efficacy, high bioavailability, and low toxicity? How to administrate treatment (systemic, peripheral, intrathecal, or local)? How to target specific cells? With these and other related questions being answered, the significance of lysosomal ion channels in pain research and pain killer development may be defined hopefully.

## Disclosures

The authors have no conflict of interest to declare.

## References

[1] Bajaj L Lotfi P Pal R Ronza AD Sharma J Sardiello M. Lysosome biogenesis in health and disease. J Neurochem 2019;148:573–89. 30092616 10.1111/jnc.14564 PMC6368902

[2] Bandell M Story GM Hwang SW Viswanath V Eid SR Petrus MJ Earley TJ Patapoutian A. Noxious cold ion channel TRPA1 is activated by pungent compounds and bradykinin. Neuron 2004;41:849–57. 15046718 10.1016/s0896-6273(04)00150-3

[3] Bandyopadhyay S Adebayo D Obaseki E Hariri H. Lysosomal membrane contact sites: integrative hubs for cellular communication and homeostasis. Curr Top Membr 2024;93:85–116. 39181579 10.1016/bs.ctm.2024.07.001 PMC12570271

[4] Basso L Altier C. Transient receptor potential channels in neuropathic pain. Curr Opin Pharmacol 2017;32:9–15. 27835802 10.1016/j.coph.2016.10.002

[5] Bautista DM Jordt SE Nikai T Tsuruda PR Read AJ Poblete J Yamoah EN Basbaum AI Julius D. TRPA1 mediates the inflammatory actions of environmental irritants and proalgesic agents. Cell 2006;124:1269–82. 16564016 10.1016/j.cell.2006.02.023

[6] Bautista DM Movahed P Hinman A Axelsson HE Sterner O Högestätt ED Julius D Jordt SE Zygmunt PM. Pungent products from garlic activate the sensory ion channel TRPA1. Proc Natl Acad Sci U S A 2005;102:12248–52. 16103371 10.1073/pnas.0505356102 PMC1189336

[7] Berliocchi L Russo R Maiarù M Levato A Bagetta G Corasaniti MT. Autophagy impairment in a mouse model of neuropathic pain. Mol Pain 2011;7:83. 22023914 10.1186/1744-8069-7-83 PMC3234188

[8] Boya P. Lysosomal function and dysfunction: mechanism and disease. Antioxid Redox Signal 2012;17:766–74. 22098160 10.1089/ars.2011.4405

[9] Cai W Li P Gu M Xu H. Lysosomal ion channels and lysosome-organelle interactions. Handb Exp Pharmacol 2023;278:93–108. 36882602 10.1007/164_2023_640

[10] Cang C Aranda K Seo YJ Gasnier B Ren D. TMEM175 is an organelle K(+) channel regulating lysosomal function. Cell 2015;162:1101–12. 26317472 10.1016/j.cell.2015.08.002

[11] Cao Q Zhong XZ Zou Y Zhang Z Toro L Dong XP. BK channels alleviate lysosomal storage diseases by providing positive feedback regulation of lysosomal Ca2+ release. Dev Cell 2015;33:427–41. 25982675 10.1016/j.devcel.2015.04.010

[12] Caspani O Zurborg S Labuz D Heppenstall PA. The contribution of TRPM8 and TRPA1 channels to cold allodynia and neuropathic pain. PLoS One 2009;4:e7383. 19812688 10.1371/journal.pone.0007383 PMC2753652

[13] Cen J Hu N Shen J Gao Y Lu H. Pathological functions of lysosomal ion channels in the central nervous system. Int J Mol Sci 2024;25:6565. 38928271 10.3390/ijms25126565 PMC11203704

[14] Chapel A Kieffer-Jaquinod S Sagné C Verdon Q Ivaldi C Mellal M Thirion J Jadot M Bruley C Garin J Gasnier B Journet A. An extended proteome map of the lysosomal membrane reveals novel potential transporters. Mol Cell Proteomics 2013;12:1572–88. 23436907 10.1074/mcp.M112.021980 PMC3675815

[15] Chen CC Cang C Fenske S Butz E Chao YK Biel M Ren D Wahl-Schott C Grimm C. Patch-clamp technique to characterize ion channels in enlarged individual endolysosomes. Nat Protoc 2017;12:1639–58. 28726848 10.1038/nprot.2017.036

[16] Chen CC Krogsaeter E Butz ES Li Y Puertollano R Wahl-Schott C Biel M Grimm C. TRPML2 is an osmo/mechanosensitive cation channel in endolysosomal organelles. Sci Adv 2020;6:eabb5064. 33177082 10.1126/sciadv.abb5064 PMC7673730

[17] Chen GL Li JY Chen X Liu JW Zhang Q Liu JY Wen J Wang N Lei M Wei JP Yi L Li JJ Ling YP Yi HQ Hu Z Duan J Zhang J Zeng B. Mechanosensitive channels TMEM63A and TMEM63B mediate lung inflation-induced surfactant secretion. J Clin Invest 2024;134:e174508. 38127458 10.1172/JCI174508 PMC10904053

[18] Chen X Wang N Liu JW Zeng B Chen GL. TMEM63 mechanosensitive ion channels: activation mechanisms, biological functions and human genetic disorders. Biochem Biophys Res Commun 2023;683:149111. 37857161 10.1016/j.bbrc.2023.10.043

[19] Chen Y Yang C Wang ZJ. Proteinase-activated receptor 2 sensitizes transient receptor potential vanilloid 1, transient receptor potential vanilloid 4, and transient receptor potential ankyrin 1 in paclitaxel-induced neuropathic pain. Neuroscience 2011;193:440–51. 21763756 10.1016/j.neuroscience.2011.06.085

[20] Cheng X Shen D Samie M Xu H. Mucolipins: Intracellular TRPML1-3 channels. FEBS Lett 2010;584:2013–21. 20074572 10.1016/j.febslet.2009.12.056 PMC2866799

[21] Cheng X Zhang X Gao Q Ali Samie M Azar M Tsang WL Dong L Sahoo N Li X Zhuo Y Garrity AG Wang X Ferrer M Dowling J Xu L Han R Xu H. The intracellular Ca²⁺ channel MCOLN1 is required for sarcolemma repair to prevent muscular dystrophy. Nat Med 2014;20:1187–92. 25216637 10.1038/nm.3611 PMC4192061

[22] Chu J Yang J Zhou Y Chen J Chen KH Zhang C Cheng HY Koylass N Liu JO Guan Y Qiu Z. ATP-Releasing SWELL1 channel in spinal microglia contributes to neuropathic pain. Sci Adv 2023;9:eade9931. 36989353 10.1126/sciadv.ade9931 PMC10058245

[23] Colacurcio DJ Nixon RA. Disorders of lysosomal acidification-the emerging role of v-ATPase in aging and neurodegenerative disease. Ageing Res Rev 2016;32:75–88. 27197071 10.1016/j.arr.2016.05.004 PMC5112157

[24] Cuajungco MP Silva J Habibi A Valadez JA. The mucolipin-2 (TRPML2) ion channel: a tissue-specific protein crucial to normal cell function. Pflugers Arch 2016;468:177–92. 26336837 10.1007/s00424-015-1732-2 PMC4715775

[25] Datta G Miller NM Afghah Z Geiger JD Chen X. HIV-1 gp120 promotes lysosomal exocytosis in human schwann cells. Front Cell Neurosci 2019;13:329. 31379513 10.3389/fncel.2019.00329 PMC6650616

[26] Davis AA Andruska KM Benitez BA Racette BA Perlmutter JS Cruchaga C. Variants in GBA, SNCA, and MAPT influence Parkinson disease risk, age at onset, and progression. Neurobiol Aging 2016;37:209 e1–e7. 10.1016/j.neurobiolaging.2015.09.014 PMC4688052 26601739

[27] Deneka D Sawicka M Lam AKM Paulino C Dutzler R. Structure of a volume-regulated anion channel of the LRRC8 family. Nature 2018;558:254–9. 29769723 10.1038/s41586-018-0134-y

[28] Dong XP Cheng X Mills E Delling M Wang F Kurz T Xu H. The type IV mucolipidosis-associated protein TRPML1 is an endolysosomal iron release channel. Nature 2008;455:992–6. 18794901 10.1038/nature07311 PMC4301259

[29] Duncan LM Deeds J Hunter J Shao J Holmgren LM Woolf EA Tepper RI Shyjan AW. Down-regulation of the novel gene melastatin correlates with potential for melanoma metastasis. Cancer Res 1998;58:1515–20. 9537257

[30] Duveau A Bertin E Boué-Grabot E. Implication of neuronal versus microglial P2X4 receptors in central nervous system disorders. Neurosci Bull 2020;36:1327–43. 32889635 10.1007/s12264-020-00570-y PMC7674530

[31] Farfel-Becker T Roney JC Cheng XT Li S Cuddy SR Sheng ZH. Neuronal soma-derived degradative lysosomes are continuously delivered to distal axons to maintain local degradation capacity. Cell Rep 2019;28:51–64 e4. 31269450 10.1016/j.celrep.2019.06.013 PMC6696943

[32] Feng T Zheng H Zhang Z Fan P Yang X. Mechanism and therapeutic targets of the involvement of a novel lysosomal proton channel TMEM175 in Parkinson's disease. Ageing Res Rev 2024;100:102373. 38960046 10.1016/j.arr.2024.102373

[33] Feng X Liu S Xu H. Not just protons: chloride also activates lysosomal acidic hydrolases. J Cell Biol 2023;222:e202305007. 37191899 10.1083/jcb.202305007 PMC10191866

[34] Feng X Zhao Z Li Q Tan Z. Lysosomal potassium channels: potential roles in lysosomal function and neurodegenerative diseases. CNS Neurol Disord Drug Targets 2018;17:261–6. 29422008 10.2174/1871527317666180202110717

[35] Fu H Bartz JD Stephens RL Jr Mccarty DM. Peripheral nervous system neuropathology and progressive sensory impairments in a mouse model of mucopolysaccharidosis IIIB. PLoS One 2012;7:e45992. 23049915 10.1371/journal.pone.0045992 PMC3457935

[36] Gauchan P Andoh T Kato A Kuraishi Y. Involvement of increased expression of transient receptor potential melastatin 8 in oxaliplatin-induced cold allodynia in mice. Neurosci Lett 2009;458:93–5. 19375484 10.1016/j.neulet.2009.04.029

[37] Gebhardt LA Kichko TI Fischer MJM Reeh PW. TRPA1-dependent calcium transients and CGRP release in DRG neurons require extracellular calcium. J Cell Biol 2020;219:e201702151. 32434221 10.1083/jcb.201702151 PMC7265312

[38] Gerndt S Chen CC Chao YK Yuan Y Burgstaller S Scotto Rosato A Krogsaeter E Urban N Jacob K Nguyen ONP Miller MT Keller M Vollmar AM Gudermann T Zierler S Schredelseker J Schaefer M Biel M Malli R Wahl-Schott C Bracher F Patel S Grimm C. Agonist-mediated switching of ion selectivity in TPC2 differentially promotes lysosomal function. Elife 2020;9:e54712. 32167471 10.7554/eLife.54712 PMC7108868

[39] Ghosh D Pinto S Danglot L Vandewauw I Segal A Van Ranst N Benoit M Janssens A Vennekens R Vanden Berghe P Galli T Vriens J Voets T. VAMP7 regulates constitutive membrane incorporation of the cold-activated channel TRPM8. Nat Commun 2016;7:10489. 26843440 10.1038/ncomms10489 PMC4742910

[40] González-Muñiz R Bonache MA Martín-Escura C Gómez-Monterrey I. Recent progress in TRPM8 modulation: an update. Int J Mol Sci 2019;20:2618. 31141957 10.3390/ijms20112618 PMC6600640

[41] Griffin CS Alvarado MG Yamasaki E Drumm BT Krishnan V Ali S Nagle EM Sanders KM Earley S. The intracellular Ca(2+) release channel TRPML1 regulates lower urinary tract smooth muscle contractility. Proc Natl Acad Sci U S A 2020;117:30775–86. 33199609 10.1073/pnas.2016959117 PMC7720193

[42] Grimm C Jörs S Saldanha SA Obukhov AG Pan B Oshima K Cuajungco MP Chase P Hodder P Heller S. Small molecule activators of TRPML3. Chem Biol 2010;17:135–48. 20189104 10.1016/j.chembiol.2009.12.016 PMC2834294

[43] Guan XJ Deng ZQ Liu J Su CF Tong BC Zhu Z Sreenivasmurthy SG Kan YX Lu KJ Chu CP Pi RB Cheung KH Iyaswamy A Song JX Li M. Corynoxine promotes TFEB/TFE3-mediated autophagy and alleviates Aβ pathology in Alzheimer's disease models. Acta Pharmacol Sin 2024;45:900–13. 38225393 10.1038/s41401-023-01197-1 PMC11053156

[44] Guo J Zeng W Jiang Y. Tuning the ion selectivity of two-pore channels. Proc Natl Acad Sci U S A 2017;114:1009–14. 28096396 10.1073/pnas.1616191114 PMC5293054

[45] Hammer P Banck MS Amberg R Wang C Petznick G Luo S Khrebtukova I Schroth GP Beyerlein P Beutler AS. mRNA-seq with agnostic splice site discovery for nervous system transcriptomics tested in chronic pain. Genome Res 2010;20:847–60. 20452967 10.1101/gr.101204.109 PMC2877581

[46] Hayashi Y Morinaga S Zhang J Satoh Y Meredith AL Nakata T Wu Z Kohsaka S Inoue K Nakanishi H. BK channels in microglia are required for morphine-induced hyperalgesia. Nat Commun 2016;7:11697. 27241733 10.1038/ncomms11697 PMC4895018

[47] Hiruma H Kawakami T. Effects of 4-aminopyridine on organelle movement in cultured mouse dorsal root ganglion neurites. J Mol Neurosci 2010;40:295–302. 19642025 10.1007/s12031-009-9219-2

[48] Hofmann L Hose D Grießhammer A Blum R Döring F Üçeyler N Dib-Hajj S Waxman S Sommer C Wischmeyer E. Characterization of small fiber pathology in a mouse model of Fabry disease. Elife 2018;7:e39300. 30328411 10.7554/eLife.39300 PMC6255391

[49] Horváth Á Tékus V Boros M Pozsgai G Botz B Borbély É Szolcsányi J Pintér E Helyes Z. Transient receptor potential ankyrin 1 (TRPA1) receptor is involved in chronic arthritis: in vivo study using TRPA1-deficient mice. Arthritis Res Ther 2016;18:6. 26746673 10.1186/s13075-015-0904-y PMC4718022

[50] Hu M Feng X Liu Q Liu S Huang F Xu H. The ion channels of endomembranes. Physiol Rev 2024;104:1335–85. 38451235 10.1152/physrev.00025.2023 PMC11381013

[51] Hu M Li P Wang C Feng X Geng Q Chen W Marthi M Zhang W Gao C Reid W Swanson J Du W Hume RI Xu H. Parkinson's disease-risk protein TMEM175 is a proton-activated proton channel in lysosomes. Cell 2022;185:2292–308.e20. 35750034 10.1016/j.cell.2022.05.021 PMC9236176

[52] Hu Z Zhang Y Yu W Li J Yao J Zhang J Wang J Wang C. Transient receptor potential ankyrin 1 (TRPA1) modulators: recent update and future perspective. Eur J Med Chem 2023;257:115392. 37269667 10.1016/j.ejmech.2023.115392

[53] Hua J Garcia de Paco E Linck N Maurice T Desrumaux C Manoury B Rassendren F Ulmann L. Microglial P2X4 receptors promote ApoE degradation and contribute to memory deficits in Alzheimer's disease. Cell Mol Life Sci 2023;80:138. 37145189 10.1007/s00018-023-04784-x PMC10163120

[54] Iftinca M Altier C. The cool things to know about TRPM8!. Channels (Austin) 2020;14:413–20. 33147416 10.1080/19336950.2020.1841419 PMC7657583

[55] Imari K Harada Y Zhang J Mori Y Hayashi Y. KCNMB3 in spinal microglia contributes to the generation and maintenance of neuropathic pain in mice. Int J Mol Med 2019;44:1585–93. 31364720 10.3892/ijmm.2019.4279

[56] Iwaki H Blauwendraat C Leonard HL Liu G Maple-Grødem J Corvol JC Pihlstrøm L Van Nimwegen M Hutten SJ Nguyen KDH Rick J Eberly S Faghri F Auinger P Scott KM Wijeyekoon R Van Deerlin VM Hernandez DG Day-Williams AG Brice A Alves G Noyce AJ Tysnes OB Evans JR Breen DP Estrada K Wegel CE Danjou F Simon DK Ravina B Toft M Heutink P Bloem BR Weintraub D Barker RA Williams-Gray CH Van de Warrenburg BP Van Hilten JJ Scherzer CR Singleton AB Nalls MA. Genetic risk of Parkinson disease and progression:: an analysis of 13 longitudinal cohorts. Neurol Genet 2019;5:e348. 31404238 10.1212/NXG.0000000000000348 PMC6659137

[57] Izquierdo C Martín-Martínez M Gómez-Monterrey I González-Muñiz R. TRPM8 channels: advances in structural studies and pharmacological modulation. Int J Mol Sci 2021;22:8502. 34445208 10.3390/ijms22168502 PMC8395166

[58] Jinn S Drolet RE Cramer PE Wong AH Toolan DM Gretzula CA Voleti B Vassileva G Disa J Tadin-Strapps M Stone DJ. TMEM175 deficiency impairs lysosomal and mitochondrial function and increases alpha-synuclein aggregation. Proc Natl Acad Sci U S A 2017;114:2389–94. 28193887 10.1073/pnas.1616332114 PMC5338534

[59] Jung J Shin YH Konishi H Lee SJ Kiyama H. Possible ATP release through lysosomal exocytosis from primary sensory neurons. Biochem Biophys Res Commun 2013;430:488–93. 23237805 10.1016/j.bbrc.2012.12.009

[60] Jung J Uesugi N Jeong NY Park BS Konishi H Kiyama H. Increase of transcription factor EB (TFEB) and lysosomes in rat DRG neurons and their transportation to the central nerve terminal in dorsal horn after nerve injury. Neuroscience 2016;313:10–22. 26601776 10.1016/j.neuroscience.2015.11.028

[61] Kalatzis V Cherqui S Antignac C Gasnier B. Cystinosin, the protein defective in cystinosis, is a H(+)-driven lysosomal cystine transporter. EMBO J 2001;20:5940–9. 11689434 10.1093/emboj/20.21.5940 PMC125690

[62] Katona I Zhang X Bai Y Shy ME Guo J Yan Q Hatfield J Kupsky WJ Li J. Distinct pathogenic processes between Fig4-deficient motor and sensory neurons. Eur J Neurosci 2011;33:1401–10. 21410794 10.1111/j.1460-9568.2011.07651.x

[63] Katsura H Obata K Mizushima T Yamanaka H Kobayashi K Dai Y Fukuoka T Tokunaga A Sakagami M Noguchi K. Antisense knock down of TRPA1, but not TRPM8, alleviates cold hyperalgesia after spinal nerve ligation in rats. Exp Neurol 2006;200:112–23. 16546170 10.1016/j.expneurol.2006.01.031

[64] Kayama Y Shibata M Takizawa T Ibata K Nakahara J Shimizu T Toriumi H Yuzaki M Suzuki N. Signaling pathways relevant to nerve growth factor-induced upregulation of transient receptor potential M8 expression. Neuroscience 2017;367:178–88. 29102663 10.1016/j.neuroscience.2017.10.037

[65] Kendall RL Holian A. The role of lysosomal ion channels in lysosome dysfunction. Inhal Toxicol 2021;33:41–54. 33627009 10.1080/08958378.2021.1876188

[66] Kim HJ Li Q Tjon-Kon-Sang S So I Kiselyov K Soyombo AA Muallem S. A novel mode of TRPML3 regulation by extracytosolic pH absent in the varitint-waddler phenotype. EMBO J 2008;27:1197–205. 18369318 10.1038/emboj.2008.56 PMC2367400

[67] Kim HJ Soyombo AA Tjon-Kon-Sang S So I Muallem S. The Ca(2+) channel TRPML3 regulates membrane trafficking and autophagy. Traffic 2009;10:1157–67. 19522758 10.1111/j.1600-0854.2009.00924.x PMC2993507

[68] Kim HK Lee SY Koike N Kim E Wirianto M Burish MJ Yagita K Lee HK Chen Z Chung JM Abdi S Yoo SH. Circadian regulation of chemotherapy-induced peripheral neuropathic pain and the underlying transcriptomic landscape. Sci Rep 2020;10:13844. 32796949 10.1038/s41598-020-70757-w PMC7427990

[69] Koivisto A Hukkanen M Saarnilehto M Chapman H Kuokkanen K Wei H Viisanen H Akerman KE Lindstedt K Pertovaara A. Inhibiting TRPA1 ion channel reduces loss of cutaneous nerve fiber function in diabetic animals: sustained activation of the TRPA1 channel contributes to the pathogenesis of peripheral diabetic neuropathy. Pharmacol Res 2012;65:149–58. 22133672 10.1016/j.phrs.2011.10.006

[70] Kwan KY Allchorne AJ Vollrath MA Christensen AP Zhang DS Woolf CJ Corey DP. TRPA1 contributes to cold, mechanical, and chemical nociception but is not essential for hair-cell transduction. Neuron 2006;50:277–89. 16630838 10.1016/j.neuron.2006.03.042

[71] Lacombe J Karsenty G Ferron M. Regulation of lysosome biogenesis and functions in osteoclasts. Cell Cycle 2013;12:2744–52. 23966172 10.4161/cc.25825 PMC3899188

[72] Lee D Hong JH. Nanoparticle-mediated therapeutic application for modulation of lysosomal ion channels and functions. Pharmaceutics 2020;12:217. 32131531 10.3390/pharmaceutics12030217 PMC7150957

[73] Li K Guo Y Wang Y Zhu R Chen W Cheng T Zhang X Jia Y Liu T Zhang W Jan LY Jan YN. Drosophila TMEM63 and mouse TMEM63A are lysosomal mechanosensory ion channels. Nat Cell Biol 2024;26:393–403. 38388853 10.1038/s41556-024-01353-7 PMC10940159

[74] Li P Hu M Wang C Feng X Zhao Z Yang Y Sahoo N Gu M Yang Y Xiao S Sah R Cover TL Chou J Geha R Benavides F Hume RI Xu H. LRRC8 family proteins within lysosomes regulate cellular osmoregulation and enhance cell survival to multiple physiological stresses. Proc Natl Acad Sci U S A 2020;117:29155–65. 33139539 10.1073/pnas.2016539117 PMC7682592

[75] Li R Lu Y Zhang Q Liu W Yang R Jiao J Liu J Gao G Yang H. Piperine promotes autophagy flux by P2RX4 activation in *SNCA*/α-synuclein-induced Parkinson disease model. Autophagy 2022;18:559–75. 34092198 10.1080/15548627.2021.1937897 PMC9037522

[76] Liao MF Lu KT Hsu JL Lee CH Cheng MY Ro LS. The role of autophagy and apoptosis in neuropathic pain formation. Int J Mol Sci 2022;23:2685. 35269822 10.3390/ijms23052685 PMC8910267

[77] Liu B Younus M Sun S Li Y Wang Y Wu X Sun X Shang S Wang C Zhu MX Zhou Z. Reply to “TRPA1-dependent calcium transients and CGRP release in DRG neurons require extracellular calcium”. J Cell Biol 2020;219:e202004017. 32434222 10.1083/jcb.202004017 PMC7265323

[78] Liu Y Mikrani R He Y Faran Ashraf Baig MM Abbas M Naveed M Tang M Zhang Q Li C Zhou X. TRPM8 channels: a review of distribution and clinical role. Eur J Pharmacol 2020;882:173312. 32610057 10.1016/j.ejphar.2020.173312

[79] Ma Y Jia T Qin F He Y Han F Zhang C. Abnormal brain protein abundance and cross-tissue mRNA expression in amyotrophic lateral sclerosis. Mol Neurobiol 2024;61:510–8. 37639066 10.1007/s12035-023-03587-2 PMC10791788

[80] Manolache A Babes A Madalina Babes R. Mini-review: the nociceptive sensory functions of the polymodal receptor transient receptor potential ankyrin type 1 (TRPA1). Neurosci Lett 2021;764:136286. 34624396 10.1016/j.neulet.2021.136286

[81] Marinelli S Nazio F Tinari A Ciarlo L D'Amelio M Pieroni L Vacca V Urbani A Cecconi F Malorni W Pavone F. Schwann cell autophagy counteracts the onset and chronification of neuropathic pain. PAIN 2014;155:93–107. 24041962 10.1016/j.pain.2013.09.013

[82] Mckemy DD Neuhausser WM Julius D. Identification of a cold receptor reveals a general role for TRP channels in thermosensation. Nature 2002;416:52–8. 11882888 10.1038/nature719

[83] Meents JE Ciotu CI Fischer MJM. TRPA1: a molecular view. J Neurophysiol 2019;121:427–43. 30485151 10.1152/jn.00524.2018

[84] Mindell JA. Lysosomal acidification mechanisms. Annu Rev Physiol 2012;74:69–86. 22335796 10.1146/annurev-physiol-012110-142317

[85] Moore C Gupta R Jordt SE Chen Y Liedtke WB. Regulation of pain and itch by TRP channels. Neurosci Bull 2018;34:120–42. 29282613 10.1007/s12264-017-0200-8 PMC5799130

[86] Murrell-Lagnado RD Frick M. P2X4 and lysosome fusion. Curr Opin Pharmacol 2019;47:126–32. 31039505 10.1016/j.coph.2019.03.002

[87] Murthy SE Dubin AE Whitwam T Jojoa-Cruz S Cahalan SM Mousavi SAR Ward AB Patapoutian A. OSCA/TMEM63 are an evolutionarily conserved family of mechanically activated ion channels. Elife 2018;7:e41844. 30382938 10.7554/eLife.41844 PMC6235560

[88] Nakagawa T Kaneko S. Roles of transient receptor potential ankyrin 1 in oxaliplatin-induced peripheral neuropathy. Biol Pharm Bull 2017;40:947–53. 28674258 10.1248/bpb.b17-00243

[89] Nakanishi-Matsui M Matsumoto N. V-ATPase a3 subunit in secretory lysosome trafficking in osteoclasts. Biol Pharm Bull 2022;45:1426–31. 36184499 10.1248/bpb.b22-00371

[90] Nassini R Gees M Harrison S De Siena G Materazzi S Moretto N Failli P Preti D Marchetti N Cavazzini A Mancini F Pedretti P Nilius B Patacchini R Geppetti P. Oxaliplatin elicits mechanical and cold allodynia in rodents via TRPA1 receptor stimulation. PAIN 2011;152:1621–31. 21481532 10.1016/j.pain.2011.02.051

[91] Nassini R Materazzi S Benemei S Geppetti P. The TRPA1 channel in inflammatory and neuropathic pain and migraine. Rev Physiol Biochem Pharmacol 2014;167:1–43. 24668446 10.1007/112_2014_18

[92] North RA. P2X receptors. Philos Trans R Soc Lond B Biol Sci 2016;371:20150427. 27377721 10.1098/rstb.2015.0427 PMC4938027

[93] Noyer L Grolez GP Prevarskaya N Gkika D Lemonnier L. TRPM8 and prostate: a cold case? Pflugers Arch 2018;470:1419–29. 29926226 10.1007/s00424-018-2169-1

[94] O'Day DH Huber RJ. Calmodulin binding proteins and neuroinflammation in multiple neurodegenerative diseases. BMC Neurosci 2022;23:10. 35246032 10.1186/s12868-022-00695-y PMC8896083

[95] Obata K Katsura H Mizushima T Yamanaka H Kobayashi K Dai Y Fukuoka T Tokunaga A Tominaga M Noguchi K. TRPA1 induced in sensory neurons contributes to cold hyperalgesia after inflammation and nerve injury. J Clin Invest 2005;115:2393–401. 16110328 10.1172/JCI25437 PMC1187934

[96] Patapoutian A Tate S Woolf CJ. Transient receptor potential channels: targeting pain at the source. Nat Rev Drug Discov 2009;8:55–68. 19116627 10.1038/nrd2757 PMC2755576

[97] Pedersen SF Okada Y Nilius B. Biophysics and physiology of the volume-regulated anion channel (VRAC)/volume-sensitive outwardly rectifying anion channel (VSOR). Pflugers Arch 2016;468:371–83. 26739710 10.1007/s00424-015-1781-6

[98] Peier AM Moqrich A Hergarden AC Reeve AJ Andersson DA Story GM Earley TJ Dragoni I Mcintyre P Bevan S Patapoutian A. A TRP channel that senses cold stimuli and menthol. Cell 2002;108:705–15. 11893340 10.1016/s0092-8674(02)00652-9

[99] Plesch E Chen CC Butz E Scotto Rosato A Krogsaeter EK Yinan H Bartel K Keller M Robaa D Teupser D Holdt LM Vollmar AM Sippl W Puertollano R Medina D Biel M Wahl-Schott C Bracher F Grimm C. Selective agonist of TRPML2 reveals direct role in chemokine release from innate immune cells. Elife 2018;7:e39720. 30479274 10.7554/eLife.39720 PMC6257821

[100] Poët M Kornak U Schweizer M Zdebik AA Scheel O Hoelter S Wurst W Schmitt A Fuhrmann JC Planells-Cases R Mole SE Hübner CA Jentsch TJ. Lysosomal storage disease upon disruption of the neuronal chloride transport protein ClC-6. Proc Natl Acad Sci U S A 2006;103:13854–9. 16950870 10.1073/pnas.0606137103 PMC1564226

[101] Ponnaiyan S Akter F Singh J Winter D. Comprehensive draft of the mouse embryonic fibroblast lysosomal proteome by mass spectrometry based proteomics. Sci Data 2020;7:68. 32103020 10.1038/s41597-020-0399-5 PMC7044164

[102] Pressey SN O'Donnell KJ Stauber T Fuhrmann JC Tyynelä J Jentsch TJ Cooper JD. Distinct neuropathologic phenotypes after disrupting the chloride transport proteins ClC-6 or ClC-7/Ostm1. J Neuropathol Exp Neurol 2010;69:1228–46. 21107136 10.1097/NEN.0b013e3181ffe742

[103] Pu S Wu Y Tong F Du WJ Liu S Yang H Zhang C Zhou B Chen Z Zhou X Han Q Du D. Mechanosensitive ion channel TMEM63A gangs up with local macrophages to modulate chronic post-amputation pain. Neurosci Bull 2023;39:177–93. 35821338 10.1007/s12264-022-00910-0 PMC9905372

[104] Qu L Lin B Zeng W Fan C Wu H Ge Y Li Q Li C Wei Y Xin J Wang X Liu D Cang C. Lysosomal K(+) channel TMEM175 promotes apoptosis and aggravates symptoms of Parkinson's disease. EMBO Rep 2022;23:e53234. 35913019 10.15252/embr.202153234 PMC9442313

[105] Riederer E Cang C Ren D. Lysosomal ion channels: what are they good for and are they druggable targets? Annu Rev Pharmacol Toxicol 2023;63:19–41. 36151054 10.1146/annurev-pharmtox-051921-013755

[106] Robinson LE Murrell-Lagnado RD. The trafficking and targeting of P2X receptors. Front Cell Neurosci 2013;7:233. 24319412 10.3389/fncel.2013.00233 PMC3837535

[107] Rohács T Lopes CM Michailidis I Logothetis DE. PI(4,5)P2 regulates the activation and desensitization of TRPM8 channels through the TRP domain. Nat Neurosci 2005;8:626–34. 15852009 10.1038/nn1451

[108] Rosato AS Tang R Grimm C. Two-pore and TRPML cation channels: regulators of phagocytosis, autophagy and lysosomal exocytosis. Pharmacol Ther 2021;220:107713. 33141027 10.1016/j.pharmthera.2020.107713

[109] Ruivo R Anne C Sagné C Gasnier B. Molecular and cellular basis of lysosomal transmembrane protein dysfunction. Biochim Biophys Acta 2009;1793:636–49. 19146888 10.1016/j.bbamcr.2008.12.008

[110] Saftig P. Physiology of the lysosome. Fabry disease: Perspectives from 5 years of FOS. Oxford: Oxford PharmaGenesis; 2006. 21290683

[111] Saftig P Klumperman J. Lysosome biogenesis and lysosomal membrane proteins: trafficking meets function. Nat Rev Mol Cell Biol 2009;10:623–35. 19672277 10.1038/nrm2745

[112] Sango K Yamanaka S Ajiki K Tokashiki A Watabe K. Lysosomal storage results in impaired survival but normal neurite outgrowth in dorsal root ganglion neurones from a mouse model of Sandhoff disease. Neuropathol Appl Neurobiol 2002;28:23–34. 11849560 10.1046/j.1365-2990.2002.00366.x

[113] Santoni G Morelli MB Amantini C Nabissi M Santoni M Santoni A. Involvement of the TRPML mucolipin channels in viral infections and anti-viral innate immune responses. Front Immunol 2020;11:739. 32425938 10.3389/fimmu.2020.00739 PMC7212413

[114] Sawicka M Dutzler R. Regulators of cell volume: the structural and functional properties of anion channels of the LRRC8 family. Curr Opin Struct Biol 2022;74:102382. 35504105 10.1016/j.sbi.2022.102382

[115] Schmidt M Dubin AE Petrus MJ Earley TJ Patapoutian A. Nociceptive signals induce trafficking of TRPA1 to the plasma membrane. Neuron 2009;64:498–509. 19945392 10.1016/j.neuron.2009.09.030 PMC2854037

[116] Schröder BA Wrocklage C Hasilik A Saftig P. The proteome of lysosomes. Proteomics 2010;10:4053–76. 20957757 10.1002/pmic.201000196

[117] Shang S Zhu F Liu B Chai Z Wu Q Hu M Wang Y Huang R Zhang X Wu X Sun L Wang Y Wang L Xu H Teng S Liu B Zheng L Zhang C Zhang F Feng X Zhu D Wang C Liu T Zhu MX Zhou Z. Intracellular TRPA1 mediates Ca2+ release from lysosomes in dorsal root ganglion neurons. J Cell Biol 2016;215:369–81. 27799370 10.1083/jcb.201603081 PMC5100290

[118] Shikha D Mahish C Sing R Chattopadhyay S Goswami C. Modulation of TRPM8 alters the phagocytic activity of microglia and induces changes in sub-cellular organelle functions. Biochem Biophys Res Commun 2023;682:56–63. 37801990 10.1016/j.bbrc.2023.09.078

[119] Silverman HA Chen A Kravatz NL Chavan SS Chang EH. Involvement of neural transient receptor potential channels in peripheral inflammation. Front Immunol 2020;11:590261. 33193423 10.3389/fimmu.2020.590261 PMC7645044

[120] Somogyi A Kirkham ED Lloyd-Evans E Winston J Allen ND Mackrill JJ Anderson KE Hawkins PT Gardiner SE Waller-Evans H Sims R Boland B O'Neill C. The synthetic TRPML1 agonist ML-SA1 rescues Alzheimer-related alterations of the endosomal-autophagic-lysosomal system. J Cell Sci 2023;136:jcs259875. 36825945 10.1242/jcs.259875 PMC10112969

[121] Souza Monteiro de Araujo D Nassini R Geppetti P De Logu F. TRPA1 as a therapeutic target for nociceptive pain. Expert Opin Ther Targets 2020;24:997–1008. 32838583 10.1080/14728222.2020.1815191 PMC7610834

[122] Spix B Jeridi A Ansari M Yildirim AÖ Schiller HB Grimm C. Endolysosomal cation channels and lung disease. Cells 2022;11:304. 35053420 10.3390/cells11020304 PMC8773812

[123] Staaf S Oerther S Lucas G Mattsson JP Ernfors P. Differential regulation of TRP channels in a rat model of neuropathic pain. PAIN 2009;144:187–99. 19446956 10.1016/j.pain.2009.04.013

[124] Story GM Peier AM Reeve AJ Eid SR Mosbacher J Hricik TR Earley TJ Hergarden AC Andersson DA Hwang SW Mcintyre P Jegla T Bevan S Patapoutian A. ANKTM1, a TRP-Like channel expressed in nociceptive neurons, is activated by cold temperatures. Cell 2003;112:819–29. 12654248 10.1016/s0092-8674(03)00158-2

[125] Suurväli J Boudinot P Kanellopoulos J Rüütel Boudinot S. P2X4: a fast and sensitive purinergic receptor. Biomed J 2017;40:245–56. 29179879 10.1016/j.bj.2017.06.010 PMC6138603

[126] Talavera K Startek JB Alvarez-Collazo J Boonen B Alpizar YA Sanchez A Naert R Nilius B. Mammalian transient receptor potential TRPA1 channels: from structure to disease. Physiol Rev 2020;100:725–803. 31670612 10.1152/physrev.00005.2019

[127] Tan SL Barri M Atakpa-Adaji P Taylor CW St John Smith E Murrell-Lagnado RD. P2X4 receptors mediate Ca(2+) release from lysosomes in response to stimulation of P2X7 and H(1) histamine receptors. Int J Mol Sci 2021;22:10492. 34638832 10.3390/ijms221910492 PMC8508626

[128] Tancini B Buratta S Delo F Sagini K Chiaradia E Pellegrino RM Emiliani C Urbanelli L. Lysosomal exocytosis: the extracellular role of an intracellular organelle. Membranes (Basel) 2020;10:406. 33316913 10.3390/membranes10120406 PMC7764620

[129] Tedeschi V Sapienza S Ciancio R Canzoniero LMT Pannaccione A Secondo A. Lysosomal channels as new molecular targets in the pharmacological therapy of neurodegenerative diseases via autophagy regulation. Curr Neuropharmacol 2025;23:375–83. 38766825 10.2174/1570159X22666240517101846 PMC12105210

[130] Toyomitsu E Tsuda M Yamashita T Tozaki-Saitoh H Tanaka Y Inoue K. CCL2 promotes P2X4 receptor trafficking to the cell surface of microglia. Purinergic Signal 2012;8:301–10. 22222817 10.1007/s11302-011-9288-x PMC3350584

[131] Trevisan G Materazzi S Fusi C Altomare A Aldini G Lodovici M Patacchini R Geppetti P Nassini R. Novel therapeutic strategy to prevent chemotherapy-induced persistent sensory neuropathy by TRPA1 blockade. Cancer Res 2013;73:3120–31. 23477783 10.1158/0008-5472.CAN-12-4370

[132] Tsuda M Shigemoto-Mogami Y Koizumi S Mizokoshi A Kohsaka S Salter MW Inoue K. P2X4 receptors induced in spinal microglia gate tactile allodynia after nerve injury. Nature 2003;424:778–83. 12917686 10.1038/nature01786

[133] Usoskin D Furlan A Islam S Abdo H Lönnerberg P Lou D Hjerling-Leffler J Haeggström J Kharchenko O Kharchenko PV Linnarsson S Ernfors P. Unbiased classification of sensory neuron types by large-scale single-cell RNA sequencing. Nat Neurosci 2015;18:145–53. 25420068 10.1038/nn.3881

[134] Venkatachalam K Wong CO Zhu MX. The role of TRPMLs in endolysosomal trafficking and function. Cell Calcium 2015;58:48–56. 25465891 10.1016/j.ceca.2014.10.008 PMC4412768

[135] Viana F. TRPA1 channels: molecular sentinels of cellular stress and tissue damage. J Physiol 2016;594:4151–69. 27079970 10.1113/JP270935 PMC4967735

[136] Wang W Zhang X Gao Q Lawas M Yu L Cheng X Gu M Sahoo N Li X Li P Ireland S Meredith A Xu H. A voltage-dependent K(+) channel in the lysosome is required for refilling lysosomal Ca(2+) stores. J Cell Biol 2017;216:1715–30. 28468834 10.1083/jcb.201612123 PMC5461029

[137] Wang X Zhang X Dong XP Samie M Li X Cheng X Goschka A Shen D Zhou Y Harlow J Zhu MX Clapham DE Ren D Xu H. TPC proteins are phosphoinositide- activated sodium-selective ion channels in endosomes and lysosomes. Cell 2012;151:372–83. 23063126 10.1016/j.cell.2012.08.036 PMC3475186

[138] Wang Y Zeng W Lin B Yao Y Li C Hu W Wu H Huang J Zhang M Xue T Ren D Qu L Cang C. CLN7 is an organellar chloride channel regulating lysosomal function. Sci Adv 2021;7:eabj9608. 34910516 10.1126/sciadv.abj9608 PMC8673761

[139] Wu B Su X Zhang W Zhang YH Feng X Ji YH Tan ZY. Oxaliplatin depolarizes the IB4(-) dorsal root ganglion neurons to drive the development of neuropathic pain through TRPM8 in mice. Front Mol Neurosci 2021;14:690858. 34149356 10.3389/fnmol.2021.690858 PMC8211750

[140] Wu JZ Zeziulia M Kwon W Jentsch TJ Grinstein S Freeman SA. ClC-7 drives intraphagosomal chloride accumulation to support hydrolase activity and phagosome resolution. J Cell Biol 2023;222:e202208155. 37010469 10.1083/jcb.202208155 PMC10072274

[141] Wu LK Agarwal S Kuo CH Kung YL Day CH Lin PY Lin SZ Hsieh DJ Huang CY Chiang CY. Artemisia leaf extract protects against neuron toxicity by TRPML1 activation and promoting autophagy/mitophagy clearance in both in vitro and in vivo models of MPP+/MPTP-induced Parkinson's disease. Phytomedicine 2022;104:154250. 35752074 10.1016/j.phymed.2022.154250

[142] Wu X Shang T Lü X Luo D Yang D. A monomeric structure of human TMEM63A protein. Proteins 2024;92:750–6. 38217391 10.1002/prot.26660

[143] Wu Y Xu M Wang P Syeda AKR Huang P Dong XP. Lysosomal potassium channels. Cell Calcium 2022;102:102536. 35016151 10.1016/j.ceca.2022.102536

[144] Xu H Delling M Li L Dong X Clapham DE. Activating mutation in a mucolipin transient receptor potential channel leads to melanocyte loss in varitint-waddler mice. Proc Natl Acad Sci U S A 2007;104:18321–6. 17989217 10.1073/pnas.0709096104 PMC2084341

[145] Xu H Ren D. Lysosomal physiology. Annu Rev Physiol 2015;77:57–80. 25668017 10.1146/annurev-physiol-021014-071649 PMC4524569

[146] Xu M Dong XP. Endolysosomal TRPMLs in cancer. Biomolecules 2021;11:65. 33419007 10.3390/biom11010065 PMC7825278

[147] Xu Y Du S Marsh JA Horie K Sato C Ballabio A Karch CM Holtzman DM Zheng H. TFEB regulates lysosomal exocytosis of tau and its loss of function exacerbates tau pathology and spreading. Mol Psychiatry 2021;26:5925–39. 32366951 10.1038/s41380-020-0738-0 PMC7609570

[148] Yamamoto K Chiba N Chiba T Kambe T Abe K Kawakami K Utsunomiya I Taguchi K. Transient receptor potential ankyrin 1 that is induced in dorsal root ganglion neurons contributes to acute cold hypersensitivity after oxaliplatin administration. Mol Pain 2015;11:69. 26567040 10.1186/s12990-015-0072-8 PMC4644342

[149] Yan H Helman G Murthy SE Ji H Crawford J Kubisiak T Bent SJ Xiao J Taft RJ Coombs A Wu Y Pop A Li D De Vries LS Jiang Y Salomons GS Van der Knaap MS Patapoutian A Simons C Burmeister M Wang J Wolf NI. Heterozygous variants in the mechanosensitive ion channel TMEM63A result in transient hypomyelination during infancy. Am J Hum Genet 2019;105:996–1004. 31587869 10.1016/j.ajhg.2019.09.011 PMC6848986

[150] Yang JM Wei ET Kim SJ Yoon KC. TRPM8 channels and dry eye. Pharmaceuticals (Basel) 2018;11:125. 30445735 10.3390/ph11040125 PMC6316058

[151] Yin Y Wu M Zubcevic L Borschel WF Lander GC Lee SY. Structure of the cold- and menthol-sensing ion channel TRPM8. Science 2018;359:237–41. 29217583 10.1126/science.aan4325 PMC5810135

[152] Zabala A Vazquez-Villoldo N Rissiek B Gejo J Martin A Palomino A Perez-Samartín A Pulagam KR Lukowiak M Capetillo-Zarate E Llop J Magnus T Koch-Nolte F Rassendren F Matute C Domercq M. P2X4 receptor controls microglia activation and favors remyelination in autoimmune encephalitis. EMBO Mol Med 2018;10:e8743. 29973381 10.15252/emmm.201708743 PMC6079537

[153] Zhang B Zhang S Polovitskaya MM Yi J Ye B Li R Huang X Yin J Neuens S Balfroid T Soblet J Vens D Aeby A Li X Cai J Song Y Li Y Tartaglia M Li Y Jentsch TJ Yang M Liu Z. Molecular basis of ClC-6 function and its impairment in human disease. Sci Adv 2023;9:eadg4479. 37831762 10.1126/sciadv.adg4479 PMC10575590

[154] Zhang M Lu H Xie X Shen H Li X Zhang Y Wu J Ni J Li H Chen G. TMEM175 mediates lysosomal function and participates in neuronal injury induced by cerebral ischemia-reperfusion. Mol Brain 2020;13:113. 32799888 10.1186/s13041-020-00651-z PMC7429711

[155] Zhang Q Li Y Jian Y Li M Wang X. Lysosomal chloride transporter CLH-6 protects lysosome membrane integrity via cathepsin activation. J Cell Biol 2023;222:e202210063. 37058288 10.1083/jcb.202210063 PMC10114921

[156] Zhang X Cheng X Yu L Yang J Calvo R Patnaik S Hu X Gao Q Yang M Lawas M Delling M Marugan J Ferrer M Xu H. MCOLN1 is a ROS sensor in lysosomes that regulates autophagy. Nat Commun 2016;7:12109. 27357649 10.1038/ncomms12109 PMC4931332

[157] Zheng W Rawson S Shen Z Tamilselvan E Smith HE Halford J Shen C Murthy SE Ulbrich MH Sotomayor M Fu TM Holt JR. TMEM63 proteins function as monomeric high-threshold mechanosensitive ion channels. Neuron 2023;111:3195–210.e7. 37543036 10.1016/j.neuron.2023.07.006 PMC10592209

[158] Zhong XZ Sun X Cao Q Dong G Schiffmann R Dong XP. BK channel agonist represents a potential therapeutic approach for lysosomal storage diseases. Sci Rep 2016;6:33684. 27670435 10.1038/srep33684 PMC5037385

[159] Zhou X Thamotharan M Gangopadhyay A Serdikoff C Adibi SA. Characterization of an oligopeptide transporter in renal lysosomes. Biochim Biophys Acta 2000;1466:372–8. 10825457 10.1016/s0005-2736(00)00201-7

[160] Zhu XC Cao L Tan MS Jiang T Wang HF Lu H Tan CC Zhang W Tan L Yu JT. Association of Parkinson's disease GWAS-linked loci with Alzheimer's disease in Han Chinese. Mol Neurobiol 2017;54:308–18. 26738859 10.1007/s12035-015-9649-5

